# 1401. Evaluating Barriers and Potential Solutions to Speaking Up About COVID-19 Symptoms: A Survey Among Nursing Home Workers

**DOI:** 10.1093/ofid/ofac492.1230

**Published:** 2022-12-15

**Authors:** Gabrielle M Gussin, Raveena D Singh, Thomas T Tjoa, Raheeb Saavedra, Sherrie H Kaplan, Susan S Huang

**Affiliations:** University of California, Irvine School of Medicine, Division of Infectious Diseases, Irvine, California; University of California, Irvine School of Medicine, Division of Infectious Diseases, Irvine, California; University of California, Irvine School of Medicine, Division of Infectious Diseases, Irvine, California; University of California, Irvine School of Medicine, Division of Infectious Diseases, Irvine, California; University of California, Irvine, School of Medicine, Irvine, California; University of California, Irvine School of Medicine, Irvine, CA

## Abstract

**Background:**

Nursing homes (NHs) are high risk settings for COVID. Staff are the primary source for introducing COVID into a NH. Preventing staff from working when ill is key to resident safety. NH staff face significant socioeconomic pressures that may influence their willingness to report COVID symptoms. Understanding the drivers behind unreported illness can inform ways to prevent working when ill.

**Methods:**

We conducted a confidential survey of 120 COVID-positive NH staff in Orange County, CA from Dec ‘20-Feb ‘22 to quantify the frequency and drivers of unreported COVID symptoms. We designed a 40-item survey to assess demographics, course of illness, symptom reporting behavior, and monetary, logistic, and emotional (stigma/fear) barriers to reporting using a 5-point Likert scale. Recruitment flyers were shared with all 70 NHs in the county and referrals were accepted from NH leadership. Participants received $50 for completing the 20-30 min phone-based survey. We calculated summary statistics, transformed all data to a 0-100 scale, assessed the reliability of each factor related to reporting at the group level using Cronbach’s alpha, and assessed discriminant validity with t-tests comparing responses among subsets expected to differ.

**Results:**

Table 1 shows participant characteristics. 49% of surveys were during the 2020-21 winter wave and 51% were during the Delta/Omicron waves, with a relatively even distribution of certified nursing assistants (CNAs), nurses, and non-frontline staff. Most cases (70%) were detected by routine testing at the NH and most (63%) had ≥1 symptom prior to their test. Only 39% disclosed their symptom to a supervisor. It is unknown how many staff would have disclosed symptoms if they were not captured during routine testing. Responses were consistent across 15 discrete factors with Cronbach alpha >0.7. Overall, fear and encouragement from supervisors were the most salient factors for speaking up about COVID symptoms (Table 2). Responses varied between the two waves and between frontline vs non-frontline workers.

**Figure:**
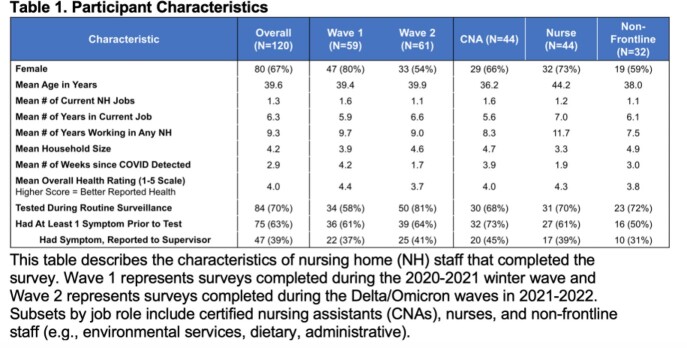

**Figure:**
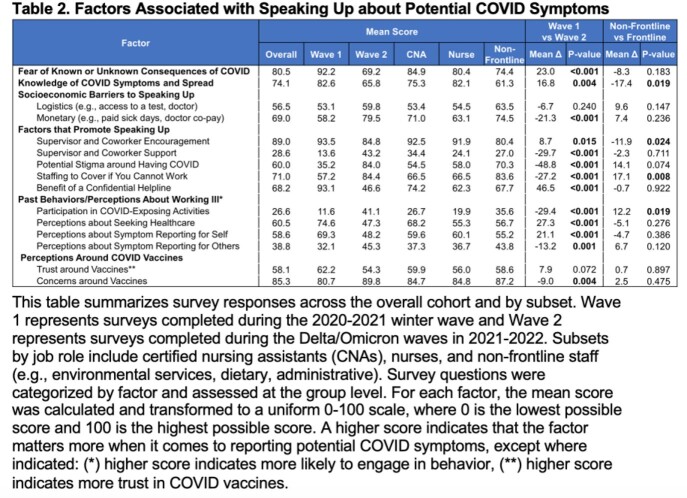

**Conclusion:**

Frequent surveillance testing of NH staff during a pandemic is critical due to many factors that drive reluctance to speak up about potential symptoms. Encouragement from supervisors to report symptoms and stay home when ill may improve NH safety.

**Disclosures:**

**Gabrielle M. Gussin, MS**, Medline: Conducted studies in which hospitals and nursing homes received contributed antiseptic and/or environmental cleaning products|Stryker: Conducted clinical studies in which hospitals and nursing homes received contributed antiseptic products|Xttrium Laboratories: Conducted clinical studies in which hospitals and nursing homes received contributed antiseptic products **Raveena D. Singh, MA**, Medline: Conducted studies in which hospitals and nursing homes received contributed antiseptic and/or environmental cleaning products|Stryker: Conducted clinical studies in which hospitals and nursing homes received contributed antiseptic products|Xttrium Laboratories: Conducted clinical studies in which hospitals and nursing homes received contributed antiseptic products **Raheeb Saavedra, AS**, Medline: Conducted studies in which hospitals and nursing homes received contributed antiseptic and/or environmental cleaning products|Stryker: Conducted clinical studies in which hospitals and nursing homes received contributed antiseptic products|Xttrium Laboratories: Conducted clinical studies in which hospitals and nursing homes received contributed antiseptic products **Susan S. Huang, MD, MPH**, Medline: Conducted studies in which hospitals and nursing homes received contributed antiseptic and/or environmental cleaning products|Molnlyke: Conducted clinical studies in which hospitals received contributed antiseptic product|Stryker: Conducted clinical studies in which hospitals and nursing homes received contributed antiseptic products|Xttrium Laboratories: Conducted clinical studies in which hospitals and nursing homes received contributed antiseptic product.

